# Supplementary material for: Recommendations for Identifying Valid Wear for Consumer-Level Wrist-Worn Activity Trackers and Acceptability of Extended Device Deployment in Children
Source: Sensors (Basel). 2022 Nov 26;22(23):9189. doi: 10.3390/s22239189 (PMC9738818; doi:10.3390/s22239189)
Supplement: Supplementary file 1 [file sensors-22-09189-s001.zip › sensors-2024927-supplementary.pdf]

Table S1. Daily average Fitbit data for boys and girls (N=137)

	**Full Sample Mean (SD) n=137**	**Girls Mean (SD) n=73**	**Boys Mean (SD) n=64**
Average minutes valid awake time per day *	860.8 (90.8)	839.0 (54.7)	885.7 (114.8)
Average resting heart rate (beats per minute) **	74 (8)	75 (7)	71 (9)
Average steps per day ****	10595 (3043)	9615 (2223	11731 (3461)
Average steps per valid awake minute ***	12.6 (3.7)	11.6 (2.8)	13.8 (4.3)
Minutes of MVPA/day****	34.6 (30.7)	19.9 (1.0)	53.1 (4.8)

MVPA= Moderate and Vigorous Physical Activity.

Note: All best practice exclusions (see Table 7 in main article) applied.

When comparing boys vs. girls.

* $p < .05$.

** $p < .01$.

*** $p < .001$

**** $p < .0001$.

Table S2. Daily average Fitbit data for weekdays vs. weekends (*N*=137 participants and 2082 days).

	**Full Sample Mean (SD) n=2082**	**Weekdays Mean (SD) n=1543**	**Weekend Days Mean (SD) n=539**
Average minutes valid awake time per day *	842.6 (124.1)	848.0 (124.4)	842.2 (110.0)
Average steps per day ***	10821 (4664)	11090 (4674)	10051 (4547)
Average steps per valid awake minute **	12.9 (5.5)	13.2 (5.4)	12.3 (5.5)
Minutes of MVPA/day***	35.4 (45.4)	37.5 (47.3)	29.7 (40.5)

MVPA= Moderate and Vigorous Physical Activity.

Note: All best practice exclusions (see Table 7 in main article) applied.

When weekday days vs. weekend days.

* $p < .05$.

** $p < .01$.

*** $p < .0001$.

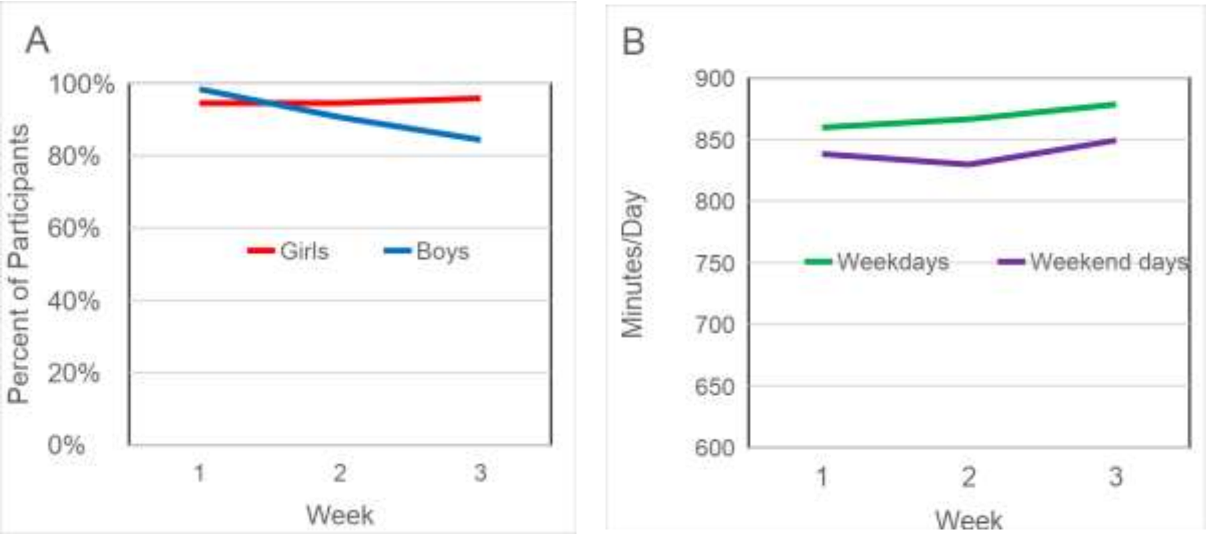

(**A**) (**B**)

Figure S1. Percent of participants with 4+ valid days of wear (including 1+ weekend day) per week (A), and average number of minutes of valid, awake wear time per day (B) across the 3-week protocol (N=137).

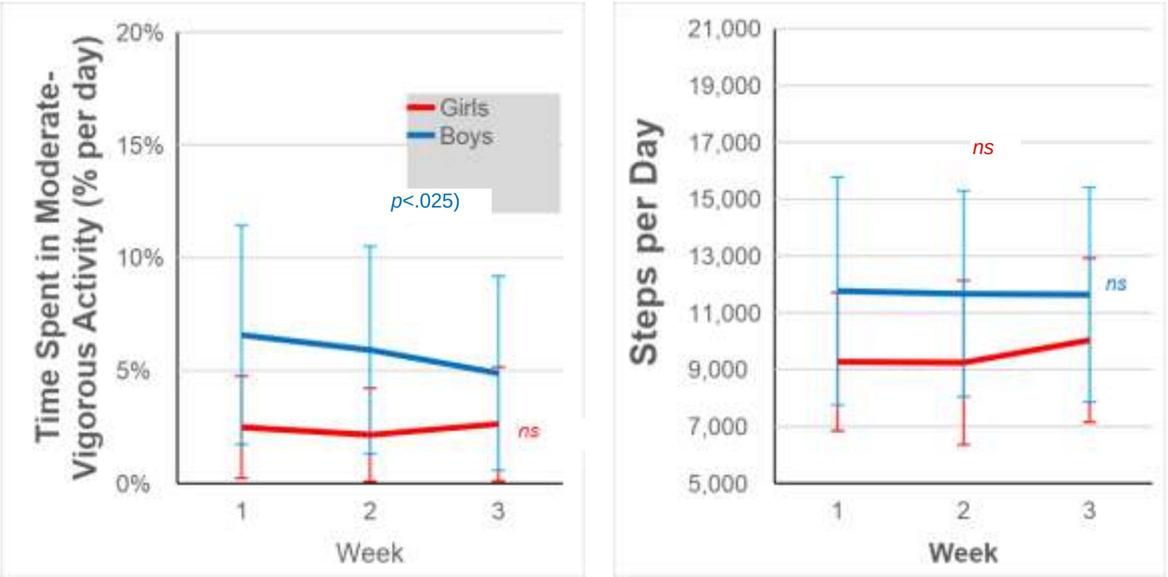

(**a**) (**b**)

Figure S2. Fitbit-recorded activity was similar during Week 1 versus Week 3 of the 3-week protocol, suggested relatively limited reactivity, with exception of boys showing a gradual reduction in moderate to vigorous activity over the 3 weeks ($p<.025$).
